# Screening Children’s Intellectual Disabilities with Phonetic Features, Facial Phenotype and Craniofacial Variability Index

**DOI:** 10.3390/brainsci13010155

**Published:** 2023-01-16

**Authors:** Yuhe Chen, Simeng Ma, Xiaoyu Yang, Dujuan Liu, Jun Yang

**Affiliations:** 1 School of Foreign Languages, Huazhong University of Science and Technology, Wuhan 430074, China; 2 Department of Psychiatry, Renmin Hospital of Wuhan University, Wuhan 430060, China; 3 Department of Pharmacy, Union Hospital, Tongji Medical College, Huazhong University of Science and Technology, Wuhan 430030, China; 4 Hubei Province Clinical Research Center for Precision Medicine for Critical Illness, Wuhan 430030, China; 5 School of Computer Science & Technology, Huazhong University of Science and Technology, Wuhan 430074, China; 6 School of Information Engineering, Wuhan University of Technology, Wuhan 430070, China

**Keywords:** facial features, phonetic features, machine learning, intellectual disability, craniofacial variability index

## Abstract

Background: Intellectual Disability (ID) is a kind of developmental deficiency syndrome caused by congenital diseases or postnatal events. This syndrome could be intervened as soon as possible if its early screening was efficient, which may improve the condition of patients and enhance their self-care ability. The early screening of ID is always achieved by clinical interview, which needs in-depth participation of medical professionals and related medical resources. Methods: A new method for screening ID has been proposed by analyzing the facial phenotype and phonetic characteristic of young subjects. First, the geometric features of subjects’ faces and phonetic features of subjects’ voice are extracted from interview videos, then craniofacial variability index (CVI) is calculated with the geometric features and the risk of ID is given with the measure of CVI. Furthermore, machine learning algorithms are utilized to establish a method for further screening ID based on facial features and phonetic features. Results: The proposed method using three feature sets, including geometric features, CVI features and phonetic features was evaluated. The best performance of accuracy was closer to 80%. Conclusions: The results using the three feature sets revealed that the proposed method may be applied in a clinical setting in the future after continuous improvement.

## 1. Introduction

Intellectual disability (ID) is a kind of generalized neurodevelopmental disorder that mostly occurs before 18 years old [1]. The intelligence and adaptive function of patients are obviously limited, including many daily social and practical skills. The prevalence of ID is estimated to be between 1% and 3% [1,2]. The lifetime costs (direct and indirect) of patients with ID is estimated to be about $1 million per person on average [3]. Common causes of ID include idiopathic genetic factors, infection or exposure to toxins during pregnancy, infant trauma at birth, malnutrition or metabolic disorders after birth and other unexplained causes [2,4,5,6].

Although there is no specific drug for the treatment of ID, there are a variety of rehabilitation programs. Early diagnosis is very important for patients’ long-term rehabilitation and to learn social skills, and can predict the prognosis of patients and reduce unnecessary diagnostic experiments [7,8].

The American Association on Intellectual and Developmental Disability defines ID through measurements in three areas: intelligence (IQ), adaptive behavior and systems of supports afforded the individual [1]. The World Health Organization (WHO) defines the severity of ID into 4 levels according to intelligence quotient (IQ) testing, i.e., mild ID (score range from 50 to 69), moderate ID (35 to 49), severe ID (20 to 34) as well as profound ID (below 20) [9].

IQ assessment needs to be conducted by highly trained doctors and is complex and time-consuming, requiring 60–90 min. In China, only the pediatrics or psychiatric departments of large hospitals have special outpatient services for intelligence assessment. However, these two departments have the most shortage of doctors, which results in the delayed diagnosis and subsequent intervention of many children. Limited by social, economic and medical restriction, insufficient attention has been paid to the diagnosis and rehabilitation of children with ID, which makes some children miss the best time for treatment. With the increase of age, there is a growing gap between these children and normal children, and there are many problems such as psychological and social adaptation, so it is very necessary to explore a simple, accurate and rapid method that can be used to screen ID.

With the advancement of artificial intelligence, related technologies have been utilized to achieve screening or preliminary diagnosis of some kinds of diseases [10]. Analyzing the subjects’ phonetic and facial features to try to achieve disease diagnosis is becoming popular [11]. People with ID may have symptoms such as severe speech delay and facial deformities such as macrostomia and/or open mouth appearance [12]. Children with ID are at higher risk for speech and language disorders. Speech and language disorders are one of the key characteristics of people with ID and can have long term negative effects on a child’s development if not treated early [13]. Some helpful clues for screening ID include delayed speech, dysmorphic features (minor anomalies), hypotonia of the extremities, general inability to do things for self, etc. [14].

Gurovich utilized computer vision and deep learning algorithms to develop a facial analysis framework, which extracted the facial features of hundreds of genetic syndromes by analyzing 2D face images. Then, the framework achieved 91% accuracy of top-10-possible-diseases in identifying 215 different genetic syndromes, which outperformed clinical experts in three different trials [15]. Abdul-Rahman analyzed 2D facial images using facial dysmorphology analysis technology, which evaluates the measurement ratio between different facial landmarks to determine whether there are deformity features. After comparing the performance of computer-based facial analysis technology against standard, manual examination in fetal alcohol spectrum disorders (FASD), the result showed that the facial dysmorphology analysis technology can potentially improve the diagnosis of alcohol-related neurodevelopmental disorder (ARND) by recognizing FASD-associated facial anomalies [16]. X-linked hypohidrotic ectodermal dysplasia is a kind of gene deficiency disease with a conspicuous facial phenotype. Hadj-Rabia designed an automated facial recognition system, which was non-invasive and achieved the diagnosis of ectodermal dysplasia for patients at all ages by analyzing their facial images [17]. 2D images could not represent patients’ facial phenotype well, so more and more researchers utilize 3D facial features to represent patients’ facial phenotype, which is a type of fundamental for disease diagnosis. Hallgrimsson explored whether syndromes can be diagnosed from 3D images of human faces, and the result showed that 3D facial imaging has considerable potential to facilitate syndrome diagnosis [18]. Gene defect diagnosis based on facial phenotype has become a new research hotspot.

Speech has been widely used in the auxiliary diagnosis of mental diseases [19]. For example, Karmele et al., proposed a non-linear multi-task method for Alzheimer’s Disease detection based on automatic speech analysis [20]. Charalambos et al., aimed to analyze whether voice quality and speech fluency distinguished people with mild cognitive impairment from healthy individuals, and the results showed that there were significant differences between people with mild cognitive impairment and healthy individuals in parameters such as cepstral peak prominence, shimmer, articulation rate and averaged speaking time [21]. In order to detect depression and predict its severity with speech assistance, Emna Rejaibi et al., proposed a MFCC-based recurrent neural network framework to perform the assessment of depression [22]. Lang He et al., combined handcrafted and deep-learned features to effectively measure the severity of depression from speech [23]. Ellen W. McGinnis et al., detected children with internalizing disorder from speech and analyzed the most discriminative speech features [24]. Meng et al., proposed a spontaneous speech-based framework, which merges mobile inverted bottleneck convolutional blocks and visual Transformer blocks, for screening mental retardation [25]. Liu et al., proposed a two stream Non-Local CNN-LSTM network to learn the features of upper body behavior and facial expression of patients to achieve preliminary screening of mental retardation [26]. In our study, the open-source tool Covara was used to extract some audio features, such as spectrum and formant, and the open-source tool OpenFace was used to extract information such as motion unit and eye gaze direction.

At present, the diagnosis of ID is based on clinical evaluation, which means clinicians need to evaluate the status of subjects through face-to-face communication. Providing an efficient and automatic screening method for ID without clinical evaluation is helpful for the diagnosis and early intervention of ID.

In this article, a benchmark data set has been established by collecting the child subjects’ video data in a clinical setting. By extracting and analyzing the features of the children’s voice and face, a new analysis system has been established for screening ID automatically. The contributions of this article are shown as follows:

- Benchmark Dataset: establishing a video data set for automatic screening ID between 6 years old and 17 years old;
- ID measurement based on CVI: By utilizing an open-source face analysis tools, high-quality 3D facial features are extracted, the subject’s facial phenotype is measured with facial features and CVI and finally, an important reference for screening ID is produced;
- ID measurement based on Voice: By extracting multiple phonetic features from the subjects’ audio, the correlation between acoustic features and ID is explored;
- Automatic Screening of ID: machine learning algorithms are utilized to analyze 3D facial features and phonetic features, and an analysis system is established to automatically screen children for ID.

However, we have to attack several challenges in order to achieve automatic ID screening for children between 7 years old and 16 years old. This article attempts to solve the related problems from the establishment of benchmark data set, extraction of high-quality 3D facial features and phonetic features, measurement of facial phenotype and the establishment of a decision-making mechanism for screening ID.

## 2. Materials and Methods

### 2.1. Dataset

The Wechsler intelligence scale for children—China revised edition (WISC-CR), which is adjusted based on WISC in order to match Chinese culture better, was used to evaluate the IQ of the subjects in clinical settings. WISC is an individually administered intelligence test for children between 7 years old and 16 years old, which can be completed without reading or writing [27]. The WISC consists of several subsets, such as Verbal Comprehension index (VCI), Perceptual Reasoning Index (PRI), Processing Speed Index (PSI) and Working Memory Index (WMI). The following four subsets were chosen to evaluate the cognitive ability of subjects, whose evaluation data constituted the benchmark dataset we used.

- Comprehension: questions about social situations or common concepts.
- Similarities: asking how two words are alike/similar.
- Picture Completion: children are shown artwork of common objects with a missing part and asked to identify the missing part by pointing and/or naming.
- Block Design: children put together red-and-white blocks in a pattern according to a displayed model. This is timed, and some of the more difficult puzzles award bonuses for speed.

Psychiatrists who have received professional training have been assigned to perform these evaluations in order to ensure the consistency of evaluation for different subjects. Meanwhile, the characteristics of WISC also guarantee the consistency of evaluation to some extent, even if the evaluation tasks have been performed by different psychiatrists [28].

During the evaluation of IQ, the subjects’ audio-visual and social demographic data were collected simultaneously. A total of 147 children were evaluated and 128 children met the experimental requirements. Among those subjects who have met the experimental requirements, there were 92 male children (71.9%). The evaluation results showed that there were 9 severe patients, 24 moderate patients, 43 mild patients and 52 normal controls. An IP camera and voice recorder were used to collect audio and video data of each subject during the evaluation. The frame rate of the video was 30, and its resolution was 640 × 480; the audio consisted of dual channels, and its sampling rate was 48 kHz. The duration of evaluation for the subjects ranged from 10 to 34 min. The sociological data collected from the subjects mainly included gender, age, height, weight, place of residence, family medical history, whether the mother had specific diseases during pregnancy, whether there were abnormal situations at birth, and whether the subject had specific diseases was as a newborn or infant. The ID label of each subject was determined after comprehensive consideration of the scale evaluation results and sociological data. Finally, the video data of 33 patients (i.e., patients with moderate and severe ID) and 37 normal controls were chosen to construct the benchmark data set. The video size of the data set is 1660 min, 520 min of which belong to 33 patients and the rest were recordings about the 37 normal controls. Table 1 shows the socio-demographic information of the subjects used to construct the dataset.

### 2.2. Analysis

**Figure 1:**
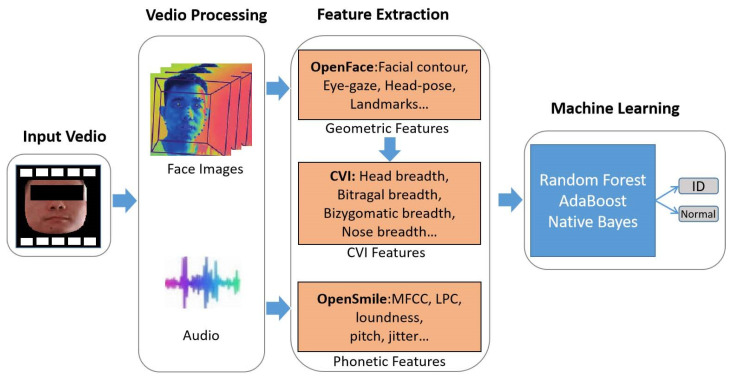
Architecture for screening ID.

**Figure 2:**
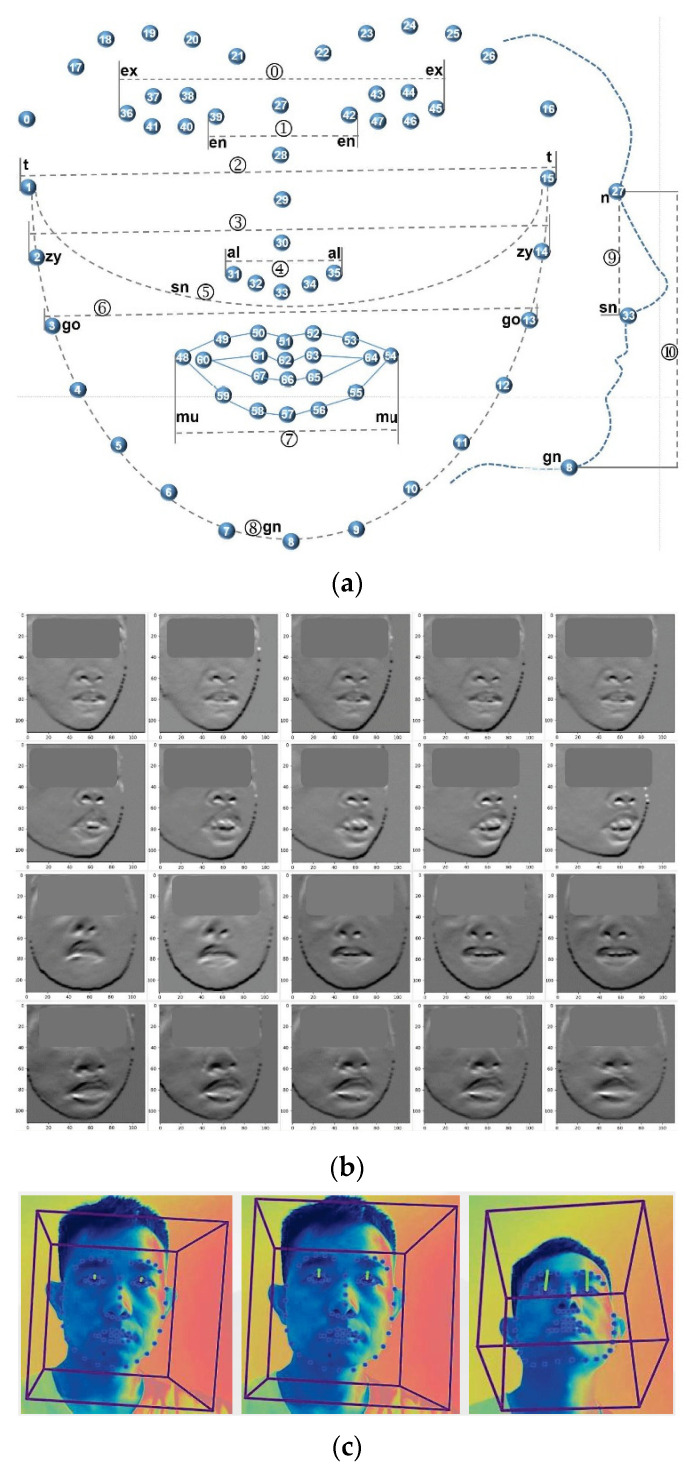
3D facial feature extraction and facial phenotype definition. (**a**) The 11 Geometric Features referenced from CVI; (**b**) Face Detection; (**c**) 3D Feature Extraction.

(1) Architecture: Figure 1 shows the architecture for screening ID. We processed WISC test videos of the subjects and extracted facial images and audio of the subjects. OpenFace and OpenSmile tools were used to extract facial features and phonetic features. Facial geometric features can be further transformed into CVI features. Furthermore, machine learning algorithms were utilized to establish a method for screening ID based on facial features and phonetic features.

(2) 3D facial features: Openface2.0 is an open-source facial behavior analysis tool, which can implement facial landmark detection, head pose estimation, facial action unit recognition and eye-gaze estimation. It has been widely used in computer vision, affective computing and human–computer interaction [29]. In this study, Openface2.0 was used to extract 3D facial features from evaluation videos, including facial contour, eye-gaze [30] and head-pose [31]. The facial features of subjects were chosen, including 3D facial landmarks of the head, to analyze the facial phenotype of subjects. Figure 2a shows the 68 facial landmarks, which represent facial contour, eye shape, nose and mouth. Each landmark was represented by an L(x, y, z) to indicate its position in 3D space. Subjects of different ages and genders possessed different scales of faces, which is not conducive to compare different faces directly. Therefore, the tool scaled the subject’s face in 3D space, and the scaling ratio is represented by p_scale; finally, the facial phenotypes of the subjects were compared at same scale. Figure 2b shows the subjects’ 2D faces images detected from videos, and Figure 2c shows the 3D facial landmarks extracted from the 2D face images. Combining the temporal information of neighbor frames, head-pose landmarks were extracted frame by frame from the videos. Neighbor frames contain subjects’ action information, which helps to extract these landmarks more accurately. The head-pose features included three different features (p_rx, p_ry, p_rz), which measures the 3D rotation degree between the head and the IP camera. In order to boost the reliability of our algorithm, we filtered the data according to the condition of confidence ≥0.98 (how confident is the tracker in current landmark detection estimate), |p_rx| ≤ 0.5, |p_ry| ≤ 0.25 and |p_rz| ≤ 0.5. Only those data meeting the above conditions, i.e., 22,602 data frames, were chosen for constructing the algorithm.

(3) Phonetic Features: Delays in speech development are common and may become more obvious when contrasted with the speech development of a sibling [14], which is a guidance for screening ID with phonetic features. Before extracting phonetic features, speech preprocessing is required, which mainly includes voice activity detection, speech enhancement and speaker-based speech segmentation. Voice activity detection can distinguish sound segments from silent segments in audio. The purpose of speech enhancement is to extract features as pure as possible from speech containing noise and improve the quality of speech. Speaker-based speech segmentation was mainly used to extracts the audio of child subjects in order to improve the effectiveness of our algorithms. The speech segmentation based on Bayesian Information Criterion was adopted in this study [32].

Every audio was processed by the OpenSmile toolkit to extract the INTERSPEECH 2010 Paralinguistic Challenge feature set, which contains 1582 low-level features [33]. The INTERSPEECH 2010 Paralinguistic Challenge feature set consists of 34 low-level descriptors (LLDs) and 34 corresponding deltas as 68 LLDS contour values, and 1428 features can be obtained from 21 functions. In addition, for the four pitch-based LLDs and their four delta coefficient contours, 19 functions are used to obtain 152 features. Finally, the number of pitch onsets (pseudo syllables) and the duration of the total input (two features) are appended [34]. The LLDS mainly include loudness, Mel Frequency Cepstral coefficients (MFCC), linear prediction coding (LPC) coefficients, jitter, shimmer and other phonetic features. Since the feature dimension is much larger than the sample size, kernel principal component analysis (KPCA) was used for feature dimension reduction, and radial basis function (RBF) was selected as the kernel function [35]. Finally, the phonetic dataset was reduced to 38 dimensions.

(4) Geometric Features and CVI: ID is often caused by gene deficiency syndrome, abnormal pregnancy, abnormal birth, brain injury, etc., which also often lead to abnormal facial phenotypes. There is a certain extent of correlation between ID and facial phenotypes, so the severity of ID for subjects can be determined through the analysis of their facial phenotypes [36]. Facial landmarks can represent the facial phenotype of subjects to some extent, and the analysis results of facial landmarks may be utilized to judge the degree of ID. Using facial landmarks to define facial phenotypic abnormalities accurately is a key factor for the performance of the algorithm. Craniofacial variability index (CVI) has been utilized to describe, characterize, and evaluate craniofacial morphology, and has been widely used in evaluating dysmorphology, diagnosing auxiliary and assessing the effect of craniofacial surgery [37]. First, 16 characteristic measurements of the head and face are obtained, and each measurement is converted into a standardized z-score. The 16 z-scores are utilized to calculate standard deviation (SD, i.e., σz), which is the CVI score. Some studies have shown that the CVI of normal people has an approximate normal distribution, and the CVI of patients with craniofacial syndrome is significantly higher than a normal person; studies also showed that utilizing a subset of 16 characteristic measurements to calculate CVI can obtain similar conclusions [38,39]. Considering all videos collected by medical professionals capture the facial information of subjects, those characteristic measurements of the face were chosen to calculate the CVI, which included 11 geometric features, as shown in Figure 2a and Table 2. Table 2 defines the formulas for calculating the 30 geometric features, and the first 11 features of which were utilized to calculate CVI. The method of calculating σz, i.e., CVI, was given in [38].

Table 3 shows the distribution of CVI for the normal group and positive group. The CVI of the positive group was higher than that of normal group, which means that it may be feasible to screen ID using the CVI. However, there is no way to judge whether a subject with a certain CVI belongs to the positive group or not. To this end, we need to build a model to screen ID utilizing all the features we obtained, including CVI.

(5) Machine Learning: The algorithms we chose to build the classification models for screening ID are Random Forest [40], AdaBoost [41] as well as Gaussian Naive Bayes [42]. All algorithms were implemented using the scikit-learn package [43] in Python.

## 3. Results

As shown in Table 4, non-CVI-related geometric features, all geometric features (including CVI) and phonetic features were utilized to establish a classification models for screening ID. Accuracy, precision, recall as well as the F1 score of the three algorithms for the three feature sets are given in Table 4. Based on geometric features, Native Bayes had the best performance compared to the other two algorithms, with an accuracy of 0.714 and F1 score of 0.715. Based on the fusion of CVI features and geometric features, Native Bayes also had the best performance, with an accuracy of 0.772 and F1 score of 0.749. Based on phonetic features, Native Bayes still had the best performance, with an accuracy of 0.796 and F1 score of 0.754. Among the three types of features, it was obvious that the best performance appeared when phonetic features were used for building the algorithms. The results also showed that phonetic features and all geometric features outperformed the non-CVI-related geometric features. In addition, the Gaussian Naive Bayes algorithm had the best performance among the three machine learning algorithms.

Next, we determined how to judge whether our proposed models are effective or not. An intuitive method is that the performance of these models should be at least better than random guesswork. In [44], a measurement was proposed to judge whether a classifier built on a particular data set performs better than random guesswork, and based on a custom data set, the classification accuracy of the proposed models must be larger than 0.646 if outperforms random guesswork. As shown in Table 4, the accuracy of the proposed models was almost 75%, which is 10 percentage points higher than 64.6%. The results showed that our work has the potential to be applied in clinical settings.

## 4. Discussion

Early diagnosis of ID is valuable because it allows the identification of children with risk, supportive counseling for parents, and potential stimulation programs for children. However, the diagnosis of ID in young children is frequently missed. Therefore, the automatic method for screening ID explored in this article provides a new perspective, which evaluates the risk of ID by analyzing subjects’ phonetic features and facial phenotype. Developmental assessment should be a part of routine pediatric care for all preschool children [45], and the method we proposed is quite suitable in terms of massive throughput of assessment and feasible assessment for very young children. A developmental pediatrician or clinical psychologist should still perform a formal assessment once ID is suspected after assessment with proposed method.

Children should be examined closely for dysmorphic features or minor abnormalities, such as unusual eyebrow patterns, eyes that are widely or closely spaced, low-set ears or abnormal palmar crease patterns. Minor abnormalities are defined as defects that have unusual morphologic features [46]. Minor physical abnormalities involve the head, eyes, ears, hands, mouth or feet, and are readily recognized even on simple examination [47]. If children have head circumferences that falls below the 5th percentile (microcephaly) or above the 95th percentile (macrocephaly), ID is suspected [12]. The presence of three or more minor abnormalities in newborns is correlated with a 90% risk of coexistent major abnormalities [48].

The study of [49] showed that the presence of ID is closely related to the level of speech ability. ID is the most common factor in speech delay [50,51]. Hearing loss and speech dystonia are common in patients with ID. The speech of children with ID is influenced not only by their cognitive impairment, but also by certain specific factors [52]. In general, the more severe the ID, the slower the acquisition of communicative speech. In children with ID, the development of language is relatively more delayed than other aspects of development [50]. Incorrect pronunciation and slurred speech are one of the clinical manifestations of speech retardation. Our findings suggest that the phonetic features of children with ID are somewhat distinguishable from normal controls.

In the future, more and more video data of new subjects should be collected, so as to improve the performance of our proposed method. Furthermore, the authors will try to establish more efficient methods from the perspective of dynamic analysis of subjects’ facial behavior patterns for screening ID. A valuable future study that we have planned is to mock the evaluation process done by a pediatrician into an applet of WeChat, which enables guardians help subjects to complete professional evaluation which usually done by pediatricians, so as to enable large-scale screening.

## Figures and Tables

**Table 1:** Characteristics of the subjects.

Variables	Number (%) or Mean (SD)
Gender	
Male	49 (70.0)
Female	21 (30.0)
Age	9.63 (3.02)
Height	139.41 (19.21)
Weight	37.00 (14.43)
BMI	18.66 (4.80)

**Table 2:** Geometric features of facial phenotype.

ID	Name	Formula	ID	Name	Formula	ID	Name	Formula
F01	ex-ex	*|P_e_08* − *P_e_42|*	F11	n-gn	*|P_f_27* − *P_f_8|*	F21	h-re	*|P_e_17* − *P_e_11|*
F02	en-en	*|P_e_14* − *P_e_36|*	F12	w-le	|*P_e_08* − *P_e_14|*	F22	w-nb	*|P_f_31* − *P_f_32* − *P_f_33* − *P_f_34* − *P_f_35|*
F03	t-t	*|P_f_01* − *P_f_15|*	F13	w-re	*|P_e_36* − *P_e_42|*	F23	h-nb	*|P_f_27* − *P_f_28* − *P_f_29* − *P_f_30|*
F04	zy-zy	*|P_f_02* − *P_f_14|*	F14	la-le	∠(*P_e_17* − *P_e_08* − *P_e_11*)	F24	a-nb	∠(*P_f_31* − *P_f_30* − *P_f_35*)
F05	al-al	*|P_f_31* − *P_f_35|*	F15	ra-le	∠(*P_e_17* − *P_e_14* − *P_e_11*)	F25	ia-lm	∠(*P_f_61* − *P_f_60* − *P_f_67*)
F06	t-sn-t	*|P_f_1* − *P_f_33* − *P_f_15|*	F16	la-re	∠(*P_e_45* − *P_e_36* − *P_e_39*)	F26	oa-lm	∠(*P_f_49* − *P_f_48* − *P_f_59*)
F07	go-go	*|P_f_03* − *P_f_13|*	F17	ra-re	∠(*P_e_45* − *P_e_42* − *P_e_39*)	F27	ia-rm	∠(*P_f_63* − *P_f_64* − *P_f_65*)
F08	mu-mu	*|P_f_48* − *P_f_54|*	F18	a-le-nb	∠(*P_e_08* − *P_e_14* − *P_e_36*)	F28	oa-rm	∠(*P_f_53* − *P_f_54* − *P_f_55*)
F09	t-gn-t	*|P_f_01* − *P_f_08* − *P_f_15|*	F19	a-re-nb	∠(*P_e_42* − *P_e_36* − *P_e_14*)	F29	a-2e	∠(*vec*(*P_e_14 − P_e_08*)*, vec*(*P_e_36 − P_e_42*))
F10	n-sn	*|P_f_27* − *P_f_33|*	F20	h-le	*|P_e_17* − *P_e_11|*	F30	a-s	∠(*vec*(*G_lx_, G_ly_, G_lz_*)*, vec*(*G_rx_, G_ry_, G_rz_*))

**Table 3:** Percentile distribution of the CVI in the two groups.

Percentile	σNor	σPos	Percentile	σNor	σPos
5th	0.404	0.577	70th	0.951	1.334
10th	0.461	0.638	75th	1.016	1.427
15th	0.503	0.685	80th	1.085	1.521
20th	0.537	0.730	85th	1.173	1.631
25th	0.567	0.776	90th	1.297	1.807
50th	0.744	0.996	95th	1.502	2.479

**Table 4:** Algorithm performance based on three different types of features.

	Accuracy	Precision	Recall	F1 Score
**Classifier**	**Geometric Features (30)**			
Random Forest	**0.715**	0.698	**0.715**	0.693
AdaBoost	0.653	0.644	0.653	0.648
Native Bayes	0.714	**0.717**	0.714	**0.715**
**Classifier**	**CVI Features + Geometric Features (31)**			
Random Forest	0.743	0.748	0.743	0.745
AdaBoost	0.715	0.714	0.715	0.715
Native Bayes	**0.772**	**0.773**	**0.772**	**0.749**
**Classifier**	**Phonetic Features (38)**			
Random Forest	0.759	0.677	0.677	0.677
AdaBoost	0.754	0.666	0.684	0.675
Native Bayes	**0.796**	**0.690**	**0.832**	**0.754**

The results with the best performance among 3 algorithms on three different feature sets are shown in bold font.

## Data Availability

The data that support the findings of this study are available on request from the corresponding author, Yang J. The data are not publicly available due to their containing information that could compromise the privacy of the research participants.
